# The effects of chronic intracortical microstimulation on neural tissue and fine motor behavior

**DOI:** 10.1088/1741-2560/12/6/066018

**Published:** 2015-10-19

**Authors:** Alexander T Rajan, Jessica L Boback, John F Dammann, Francesco V Tenore, Brock A Wester, Kevin J Otto, Robert A Gaunt, Sliman J Bensmaia

**Affiliations:** 1 Committee on Computational Neuroscience, University of Chicago, Chicago, IL, USA; 2 Department of Organismal Biology and Anatomy, University of Chicago, Chicago, IL, USA; 3 Research and Exploratory Development Department, Johns Hopkins University Applied Physics Laboratory, Laurel, MD, USA; 4 J Crayton Pruitt Family Department of Biomedical Engineering, University of Florida, Gainesville, FL, USA; 5 Department of Physical Medicine and Rehabilitation, University of Pittsburgh, Pittsburgh, PA, USA

**Keywords:** histopathology, neuronal density, neuronal damage, somatosensory cortex, neuroprosthetics, sensory feedback

## Abstract

**Objective.:**

One approach to conveying sensory feedback in neuroprostheses is to electrically stimulate sensory neurons in the cortex. For this approach to be viable, it is critical that intracortical microstimulation (ICMS) causes minimal damage to the brain. Here, we investigate the effects of chronic ICMS on the neuronal tissue across a variety of stimulation regimes in non-human primates. We also examine each animal’s ability to use their hand—the cortical representation of which is targeted by the ICMS—as a further assay of possible neuronal damage.

**Approach.:**

We implanted electrode arrays in the primary somatosensory cortex of three Rhesus macaques and delivered ICMS four hours per day, five days per week, for six months. Multiple regimes of ICMS were delivered to investigate the effects of stimulation parameters on the tissue and behavior. Parameters included current amplitude (10–100 *μ*A), pulse train duration (1, 5 s), and duty cycle (1/1, 1/3). We then performed a range of histopathological assays on tissue near the tips of both stimulated and unstimulated electrodes to assess the effects of chronic ICMS on the tissue and their dependence on stimulation parameters.

**Main results.:**

While the implantation and residence of the arrays in the cortical tissue did cause significant damage, chronic ICMS had no detectable additional effect; furthermore, the animals exhibited no impairments in fine motor control.

**Significance.:**

Chronic ICMS may be a viable means to convey sensory feedback in neuroprostheses as it does not cause significant damage to the stimulated tissue.

## Introduction

The loss of sensorimotor function has devastating consequences on quality of life. In an attempt to restore motor function, anthropomorphic robotic arms are being developed, as are algorithms to control these arms using signals from the brain [1]. Previously, signals from the motor cortex of tetraplegic patients have been used to control a robotic limb to perform motor tasks [2, 3]. While this is a remarkable accomplishment, upper-limb neuroprostheses may not be clinically viable until they include somatosensory feedback [1, 4]. Indeed, tactile and proprioceptive feedback is critical for grasping and manipulating objects and performing activities of daily living [5]. Furthermore, somatosensation plays a critical role in emotional communication and in the embodiment of our limbs. One approach to providing somatosensory feedback is to stimulate neurons in somatosensory cortex through chronically implanted microelectrodes. Intracortical microstimulation (ICMS) can elicit somatosensory percepts that can be used to guide behavior by primates [4, 6–11] and rodents [12–15]. However, to be clinically relevant, ICMS must be demonstrated to be safe and robust over years or decades.

Previous studies have suggested that repetitive electrical stimulation may damage the brain. Indeed, long term ICMS has been shown to cause dynamic changes in electrode impedance, an assay of the tissue/electrode interface [16, 17], and to lead to higher stimulation thresholds [18, 19], depression of neuronal excitability [20], and neuronal loss [21, 22]. Importantly, neuronal loss has been reported at or below stimulation parameters that may be required to elicit behaviorally relevant responses [21]. We extend these previous findings by investigating the effects of chronic ICMS on neuronal tissue over a wide range of stimulation conditions. Specifically, we chronically delivered ICMS, spanning a range of stimulation parameters, to the primary somatosensory cortex (S1) of non-human primates (NHPs) over a period of six months. A range of histopathological assays were performed on slices of cortex including tissue near the tips of both stimulated and unstimulated electrodes. These slides were analyzed in three stages: first, a histopathologist examined each slide and described any large-scale changes in the tissue; second, the histopathologist qualitatively examined tissue around electrode tips and rated the damage in each sample (initially blinded to the stimulation regime to which the electrode had been subjected); third, a quantitative analysis on the neural density around a subset of electrode tips was performed. By carefully evaluating differences in tissue damage between stimulated and non-stimulated tissue, we determined how much damage occurred as a result of stimulation beyond that sustained as a result of implantation and explantation, and assessed the degree to which damage, if any, differed across stimulation regimes. As a further assay of the effects of chronic ICMS, we assessed its consequences on fine motor control by evaluating the animals’ ability to perform precision grips. We found that, while the implantation and residence of the arrays caused significant tissue damage, chronic ICMS delivered over the span of months caused minimal additional tissue damage and no detectable behavioral deficits over the range of parameters tested.

## Methods

All procedures were approved by the University of Chicago Institutional Animal Care and Use Committee (IACUC), the Animal Care and Use Review Office (ACURO), and complied with the guidelines set by the Association for Assessment and Accreditation of Laboratory Animal Care (AAALAC) International. Furthermore, experiments were conducted under Good Laboratory Practices (GLP, Code of Federal Regulations, Title 21).

### Arrays

**Figure 1.**
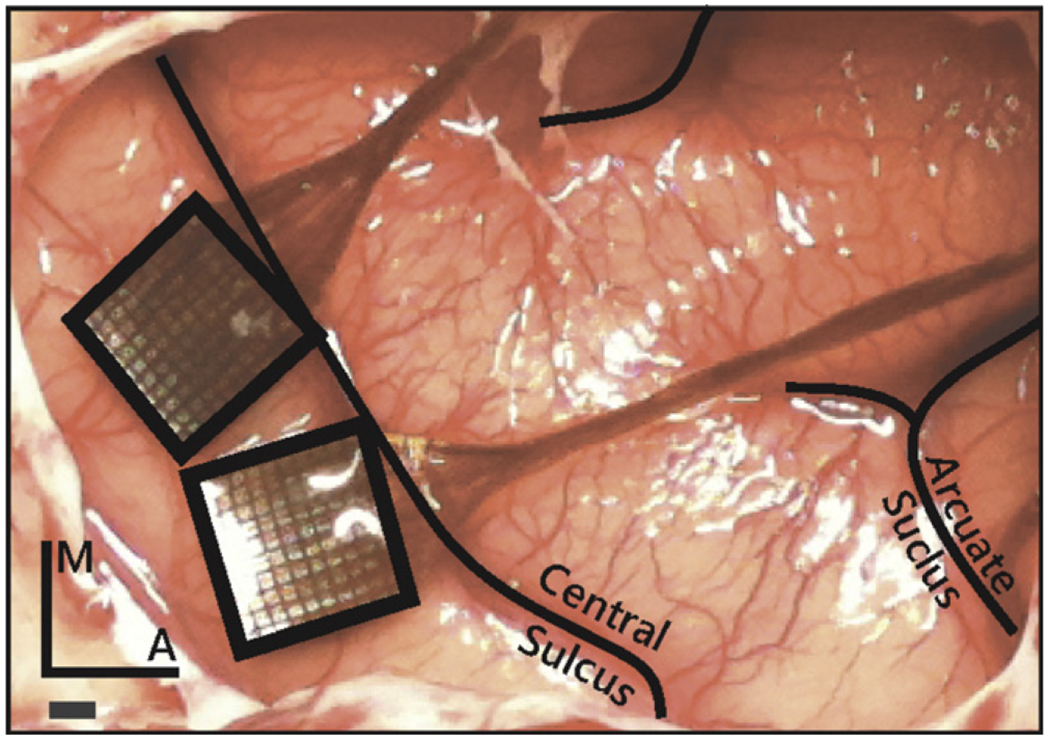
Placement of the Utah electrode arrays in one NHP. The arrays were placed in the postcentral gyrus closely abutting the central sulcus to target the hand representations in areas 1 and 2, just posterior and medial to the termination of the intraparietal sulcus. Scale bar = 1 mm.

ICMS was delivered to the primary somatosensory cortex via Utah Electrode Arrays with Cereport connectors (UEAs, Blackrock Microsystems, Salt Lake City, UT). Electrodes were 1.5 mm in length and their tips were coated with a sputtered iridium oxide film (SIROF) using the standard process [16, 23]. The electrode shaft was insulated with parylene-C along its length, with the exception of the tip, which had a targeted exposure length of 50 *μ*m. Electrode impedances were measured to be between 10 and 80 kΩ prior to implantation. Each NHP was implanted with two UEAs: one posterior and medial to the other (figure 1). We verified that receptive fields were located in the hand representation by monitoring multi-unit activity of each electrode in each UEA through speakers while palpating the hand.

### Subjects

Three rhesus macaques (two male, one female) were each surgically implanted with two UEAs in the hand representation of somatosensory cortex. Two of the NHPs were research naive prior to the study, and the third was involved in a study that did not involve the brain. All NHPs were between 6 and 8 years of age at the date of implantation.

### Surgical implantation

Having been administered atropine preoperatively (0.04 mg kg^−1^, IM), NHPs were anesthetized with a mix of ketamine hydrochloride (2–3 mg kg^−1^, IM) and dexmedetomidine (75 *μ*g kg^−1^, IV), placed in a stereotaxic instrument (Kopf Instruments, Tujunga, CA), and intubated. Anesthesia was maintained with isoflurane (1–3%). IV fluids and remifentanil (0.1–0.5 *μ*g kg^−1^ min^−1^) were delivered throughout the procedure. Two UEAs were implanted using standard methods [6, 24–27] in the hand representation of primary somatosensory cortex (areas 1 and 2), identified in each case based on stereotaxic coordinates (approximately 6 mm anterior and 22 mm lateral in ear-bar-based coordinates), and adjusted to anatomical landmarks (figure 1). Indeed, we have found the hand representation to be consistently located medial and posterior (following the central sulcus) to where the intraparietal sulcus curls towards the central sulcus.

### Stimulation protocol

**Figure 2.**
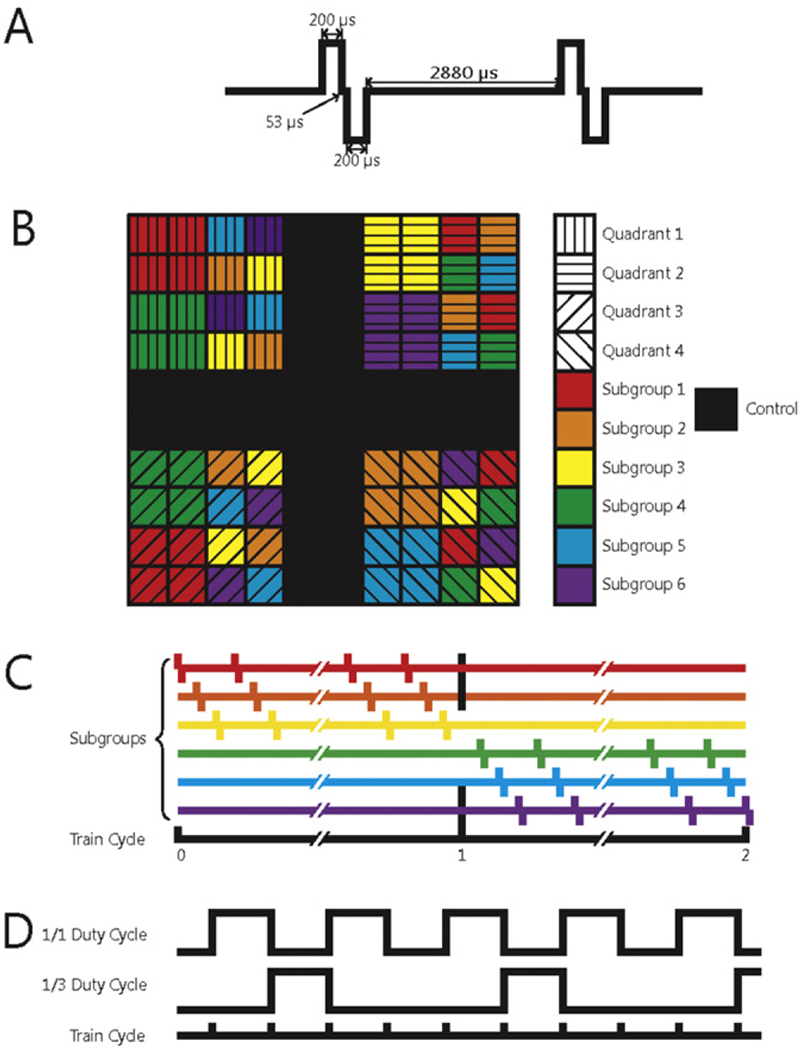
(A) Schematic of two charge-balanced stimulation pulses at 300 Hz. (B) Electrode arrangement in each array. Each quadrant of electrodes was subjected to a different regime of stimulation. Black squares denote control electrodes. Arrays were divided into subgroups designed to control the amount of charge that was simultaneously delivered to the subject. Within a given quadrant, all subgroups were stimulated with the same stimulation parameters. (C) Different stimulation regimes were also interleaved to distribute the current as evenly over time as possible. (D) Interleaving of the 1/1 (50%) and 1/3 (25%) duty cycles.

Each NHP was subjected to a 4-hour block of ICMS, 5 days per week (at the same time each day), for a period of six months (not including a one-week break during the winter holidays) beginning 9–11 weeks after implantation of the arrays. We report data from the time after onset of stimulation, not the time after array implantation. ICMS trains consisted of anodal phase-leading symmetrical pulses, (manufacturer default), with phases lasting 200 *μ*s and an inter-phase delay of 53 *μ*s, delivered at a frequency of 300 Hz using a CereStim R96 (Blackrock Microsystems Inc., Salt Lake, City, UT) (figure 2(A)). All stimulation pulses were delivered in a monopolar configuration with the titanium pedestal acting as the return electrode. Each UEA was divided into four non-contiguous quadrants, each receiving a different stimulation regime. Each quadrant consisted of a 4 × 4 grid of 16 electrodes at the corners of the 100-electrode array. Quadrants were separated by rows or columns, two electrodes wide, which received no stimulation (control electrodes) (figure 2(B)). Each quadrant was further divided into two groups of four electrodes and four groups of two electrodes (figure 2(B)). Stimulation was delivered in six asynchronous sets, each containing one group from each of the four quadrants (figure 2(C)). While electrodes within each quadrant were subjected to pulse trains with the same parameters, the subgroups defined which electrodes were activated synchronously so that a maximum of 12 electrodes per array were simultaneously activated at any given time. The goal of this grouping strategy was to limit the amount of charge that was instantaneously injected in a localized region of tissue because we found that high levels of synchronous current resulted in rhythmic muscle contractions.

Three parameters varied across stimulation regimes: charge amplitude, duty cycle, and train duration (figure 2). Charge amplitudes—10 *μ*A (2 nC/phase), 20 *μ*A (4 nC/phase), and 100 *μ*A (20 nC/phase)—were selected to span a range that have been shown to elicit a range of sensations [6] and were deemed based on previous studies to be safe [21]. Detection thresholds in primary somatosensory cortices of primates range in amplitude from 20–40 *μ*A (4–8 nC/phase) [6], but lower thresholds can be achieved by simultaneously stimulating through multiple electrodes under some circumstances [28] but not others [29]. Thus, 10 and 100 *μ*A amplitudes represented the lower and upper extremes of ICMS respectively and 20 *μ*A served as a near-threshold stimulus. These stimulus amplitudes correspond to charge densities of 0.1, 0.2 and 1.0 mC cm^−2^ assuming (per the manufacturer) that the electrodes have an exposed area of ~2000 *μ*m^2^. Both charge density and charge per phase are important as it has been understood for some time that these parameters play synergistic roles in shaping the behavior of the electrode-tissue interface [30, 31]. While electrode tip exposure can vary somewhat [16], charge per phase and charge density were below the damage threshold for SIROF electrodes (see figure 14 in [16]). Duty cycle was included as a parameter because different stimulation duty cycles have been shown to lead to different tissue responses [21]. The duty cycles were either 1:1 or 1:3. Pulse train durations were either 1 or 5 s (figure 2(C)), to span the range of what might occur during object manipulation with a prosthetic hand.

### Behavioral assessment

In order to provide a metric of the functional consequences of the long-term ICMS performed in this study, NHPs were tested on a grasping task after each stimulation session using small (<1 cm, e.g. raisin), medium (2–3 cm, e.g. grape) and large (>4 cm, e.g., apple slice) treats. Performance on each of the grasps was documented each day. Failure to grasp objects could indicate damage to somatosensory cortex based on the findings that small lesions within the fingertip regions of S1 can lead to difficulties in performing dexterous tasks [32].

### Histology and microscopy

NHPs were first anesthetized with ketamine/dexmedetomidine and isoflurane then sacrificed with a lethal dose of Pentobarbitol (100 mg Kg^−1^ IP). The descending aorta was clamped, and the NHP was transcardially perfused with heparinized phosphate buffered saline, followed by 10% neutral buffered formalin. Whole brains were sent to Charles River Laboratories (CRL), Pathology Associates (Durham, NC) for qualitative histopathological evaluation under GLP controls.

Formalin fixed, paraffin embedded tissue sections overlapping with the locations of electrode implantation were sectioned coronally at 5 *μ*m, mounted on glass slides, and then stained. Seven stains and biomarkers were used to assess tissue health around the electrodes. Neuronal nuclei (NeuN) was used as a marker of the presence of neuronal cell bodies: a reduction in NeuN density compared to the control tissue (around unstimulated electrodes or unimplanted tissue) indicates damage. Fluoro-jade B staining was used to detect degenerating neurons. Glial fibrillary acidic protein (GFAP) was used as a marker for astrocytes; an increase in the density of GFAP indicates an increase in reactive astrocytes near the electrode. Ionized calcium binding adapter molecule 1 (Iba1) is a calcium-binding protein that is specifically expressed in microglia in the brain. An increase in Iba1 expression in microglia indicates neuroinflammation, infection, degeneration, or ischemia. Hematoxylin and Eosin (H&E), luxol fast blue (LFB) and cresyl violet (CV) were used to stain nuclei and myelin and to identify anatomical landmarks, laminae, and astrocytic scars.

### Tissue slide evaluation

Tissue slides were evaluated by a board certified veterinary pathologist using light microscopy for any evidence of morphological changes and lesions in the somatosensory cortex. First, a gross histopathological analysis was performed, comparing implanted tissue with unimplanted tissue. Second, slides were scanned at a high resolution using an Aperio ScanScope slide scanner (Buffalo Grove, IL) and the scanned tissue was used to reconstruct the position of the array in the brain (see below). This reconstruction allowed us to identify the location of each electrode tip in the tissue and thus the stimulation regime to which that patch of tissue was subjected. Note that sections stained with fluoro-jade B and GFAP were examined but not scanned. As a result, they could not be not used in the three dimensional reconstructions or, therefore, in the side-by-side comparison of control electrodes and electrodes with specific stimulation regimes (since the 3D reconstruction was necessary to determine to which stimulation regime tissue on each slide was subjected). They were used, however, in the gross histopathological analysis. Third, the histopathologist visually examined the tissue surrounding each identified electrode tip (but blinded to the stimulation regime to which it had been subjected) and assigned ratings based on the quality of damage. These qualitative assessments were used to make histopathological comparisons between tissue samples that experienced different stimulation regimes. Fourth, we verified the qualitative assessment of neural density by performing a quantitative assessment in a subset of samples.

### Three-dimensional reconstructions

**Figure 3.**
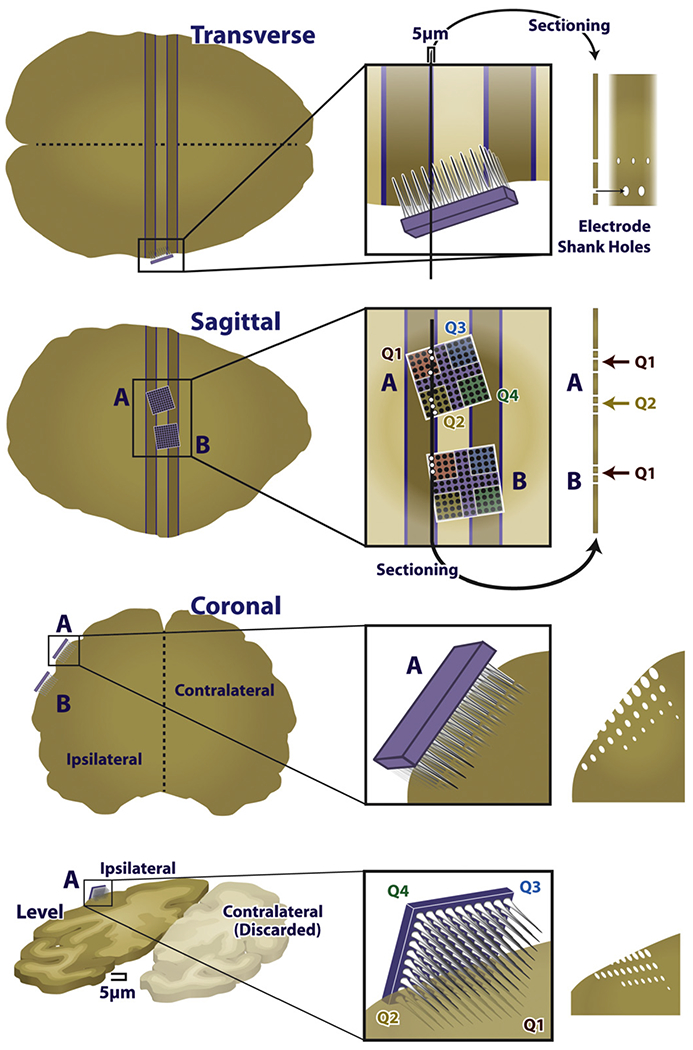
Diagram of the reconstruction approach. Example two-dimensional image of the three-dimensional reconstruction. Note the electrodes piercing the plane of the tissue, through the ellipsoid gaps in the tissue.

We focused the histopathological analysis on the tissue within 250 *μ*m of electrode tips. To identify these regions within each section, we matched patterns of lesions in each slice with electrodes on the array that caused those lesions, and then identified regions that were near a specific electrode tip. To this end, we reconstructed, based on scanned images of the slides, the position of the array in the tissue, matching individual tissue lesions with individual electrodes. To achieve this reconstruction, we aligned the two-dimensional tissue sections with three-dimensional models of UEAs (Adobe Illustrator; Google SketchUp, Pro/Engineer) (figure 3). Given the known (approximate) location of the slice relative to the array, the layout and number of insertion tracks produced from each array, and the orientation of the array in the tissue, we were able to reconstruct the position of each slice with respect to the array. Insertion tracks were noted when there was a round or oblong hole in the tissue that could not be attributed to vasculature, at an appropriate distance from nearby insertion tracks, and shaped similarly to nearby insertion track holes. These reconstructions were performed independently by two investigators, and the same results were obtained from both analyses. We then identified 79 regions of tissue that were within 250 *μ*m of the electrode tip and focused all histopathological analysis on these regions. Based on these reconstructions, we were able to unambiguously determine the stimulation paradigm to which each region of tissue was exposed.

### Histopathological assessment

Images of tissue surrounding each identified electrode tip were captured, their order randomized, and sent to CRL for a ‘blind’ qualitative histopathological assessment of the condition of the tissue. The histopathologist performing the assessment did not know from which NHP the tissue originated, nor the stimulation regime that had been delivered through the nearby electrode. The histopathologist examined the neuroparenchyma adjacent to the electrode tips for damage, including but not limited to neuronal necrosis or loss, gliosis and/or inflammation, astrocytic scarring, and pigmentation. After this examination, the stimulation condition was revealed, and a comparison of the amount of damage based on different stimulation regimes was performed. The images were graded based on four criteria: presence of insertion track; amount of gliosis; astrocytic scarring; and pigmentation. Other histopathological conditions were not evident in the tissue.

Ongoing neuronal degeneration and necrosis were diagnosed by the observation of fluoro-jade B positive neurons. Neuronal loss was evaluated by comparing the density of neurons near an electrode tip sample to adjacent intact tissue.

Gliosis was diagnosed when an increase in the number of microglia was observed (based on Iba1-stained slides). Gliosis was characterized as ‘minimal’ when very few glial cells were present, and were restricted to an area of tissue with a radius of 60 *μ*m or less. ‘Mild’ was assigned when larger numbers of glial cells were present, and formed a small nodular cluster surrounding the insertion track, extending into the adjacent parenchyma with a radius of 60–135 *μ*m. Glial scarring greater than ‘mild’ (that is, ‘moderate’ or ‘marked’) was not observed in the tissue samples. These ratings were based on standard pathology rating scales [33].

Astrocytic scars are bundles of astroglial fibers that typically surround insertion tracks [34–36] (based on primarily on H&E-stained slides). These fibers respond to the presence of foreign material by increasing the number and size of their cellular processes. Astrocytic scarring was judged on a scale analogous to that of gliosis.

Pigmentation refers to the presence of golden-brown pigment in glial cells surrounding electrode tracks. This discoloration might stem from a localized hemorrhage caused by the array implantation [35].

In order to quantify differences in tissue response across stimulation regimes, each sample was assigned a numerical score based on gliosis and astrocytic scarring. A score of 0 was ascribed when no damage was apparent, a score of 1 corresponded to minimal damage, and a score of 2 corresponded to mild damage. The scores for each stimulation regime were then compared to the control scores using an 8 × 3 Fisher–Freeman–Halton exact test, which is an extension of a 2 × 2 Fisher’s exact test that is applicable to multiple stimulation conditions (in this case eight) and outcome categories (in this case three: ‘none,’ ‘minimal,’ and ‘mild,’), and can be used with small sample sizes [37].

To validate the qualitative assessments of neuronal density provided by the histopathologist, we performed a quantitative analysis of density on a subset of samples. Specifically, we counted the number of neurons within concentric annuli defined by radii of 50, 100, and 150 *μ*m from the electrode tips identified in NeuN-stained tissue. Neuronal densities were normalized with respect to unimplanted tissue sampled from the same neuronal layers within 1–4 mm from the edge of the array on the same slice of tissue. This analysis was carried out on 9 NeuN-stained samples that had been subjected to 20 *μ*A (across duty cycles and durations) and 9 NeuN-stained samples with unstimulated electrode tips. If a given annulus was missing more than 50% of the tissue, the data was removed from the analysis (this occurred only in five 50 *μ*m circles: two controls, and three stimulated; no 100 or 150 *μ*m annuli were excluded). We could not perform this analysis on other stimulation conditions given the small number of slides stained with NeuN for those conditions.

## Results

**Figure 4.**
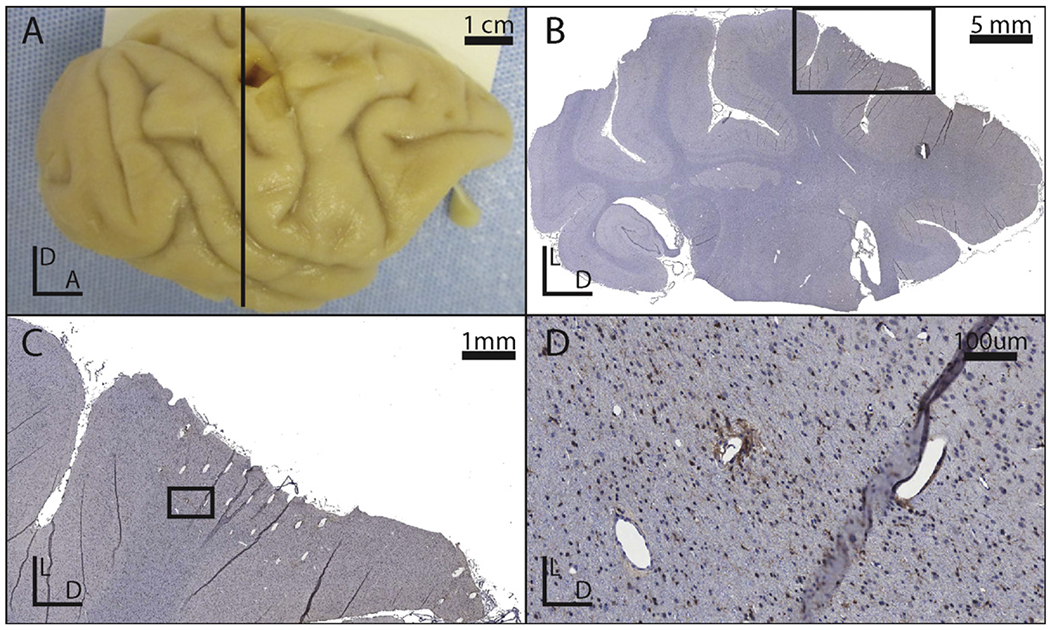
(A) Photograph of the right hemisphere of an explanted brain. (B) Picture of a coronal slice with Iba1 stain from the brain show in panel A. The black box indicates the region shown in panel C. (C) Close-up of the explanted tissue immediately deep to an implanted site. The black box indicates the region shown in panel D. (D) A close up of a lesion associated with an electrode tip. Note that the holes in the tissue which do not match the orientation of the other electrode tracks are likely vasculature.

Two UEAs were implanted between the central and intraparietal sulci, within the hand representation in areas 1 and 2 of primary somatosensory cortex of the right hemisphere (figure 1). After six months of chronic ICMS, the NHPs were perfused, and their brains were removed with the arrays and dura mater intact. After explantation of the arrays, depressions were visible in the cortex that corresponded to the shape, size, and orientation of the arrays (figure 4(A)).

### Effects of implantation and residence of arrays

The findings from gross histopathological analysis were similar across all NHPs and arrays. The tissue under all arrays was minimally to moderately atrophied. The remaining cortical thickness was usually approximately 70% of normal, adjacent tissue (range: 50–80%). The atrophy typically resulted from a partial loss of neurons from all cortical layers, and was never focused near electrode tips. Two implantations resulted in atrophy that was focused in (but not limited to) specific cortical layers: layers I and II in one array in one animal, and layer III in another array in a different animal.

In two animals, a very small number of fluoro-jade B positive neurons were present in the superficial cortex immediately deep to the array, adjacent to the interface between the array and the cerebral cortex. These neurons are indicative of ongoing neuronal degeneration and necrosis. No fluoro-jade B positivity was detected in the third animal, or in the deep cortex at the site of stimulation of any animal.

Insertion tracks from the microelectrodes were evident in all sections deep to all of the electrode arrays (figure 4(B)). Although all arrays were implanted such that the electrodes were normal to the cortical surface, the angle of the electrode tracks relative to the sectioned tissue varied according to the plane of section. More superficial tracks were larger in diameter and wedge or ovoid in shape (figure 4(C)), whereas deeper tracks were smaller in diameter and tended to be circular rather than ovoid (figure 4(D)).

Morphological changes near the electrode tip interface were localized to the immediately adjacent parenchyma, and consisted of increased number of hypertrophied astrocytes (with numerous cellular processes) and minimal aggregates of microglial cells lining the perimeter of the electrode insertion tracks. Intact neurons typically remained immediately adjacent to the tips (and were intertwined with the hypertrophied astroglial processes) suggesting the presence of astroglia did not preclude the presence of healthy neurons. No evidence of ongoing neuronal degeneration was present in the deep cortex adjacent to the electrode tips in any animal.

For the gross histopathology, the histologist was unable to identify with certainty whether individual electrodes received stimulation. However, in several slides, a sufficient number of electrode tracks were visible that these would necessarily span both stimulated and unstimulated electrodes. In these cases, the histologist found no morphological differences in the adjacent parenchyma. Thus, while the implantation and residence of the arrays in cortex did cause significant damage, the initial, gross histopathological assessment suggested that stimulation did not contribute additional damage.

### Qualitative assessment of the effects of stimulation

Next, we describe the changes to the cortical tissue near electrode tips, described by the histopathologist, grouping these descriptions by stimulation regime, which was only revealed after the assessment was made.

**Figure 5.**
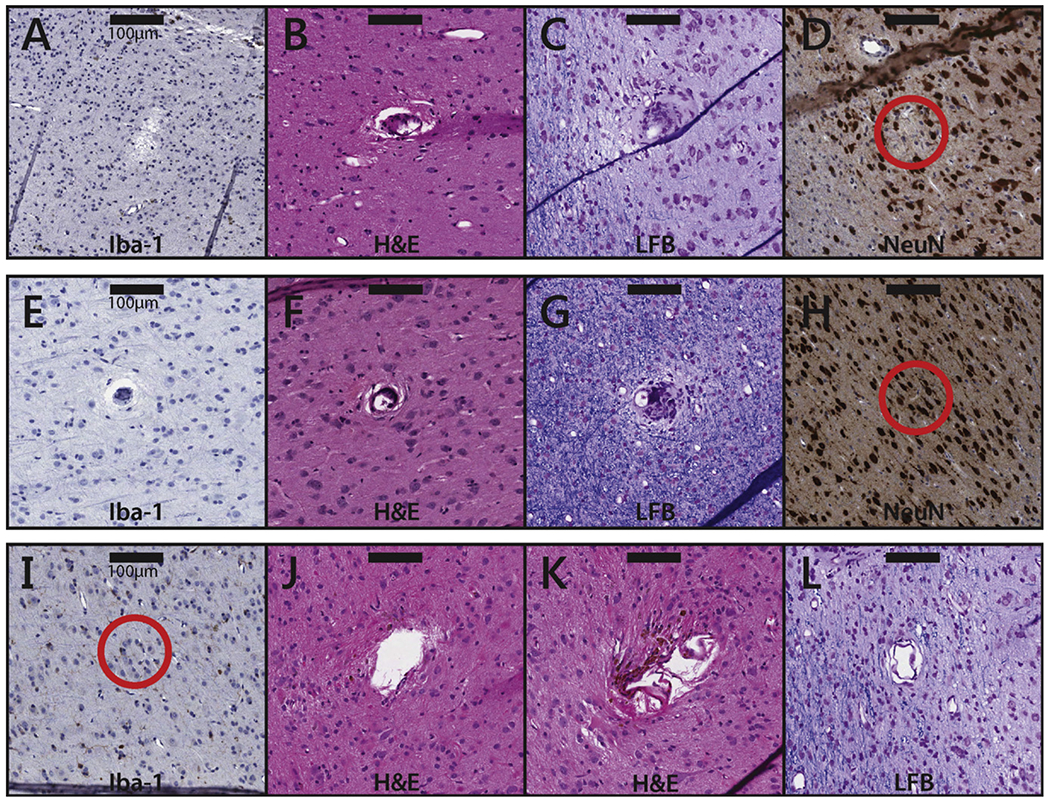
Tissue samples near electrode tips subjected to different conditions. (A)–(D) Samples near electrode tips in unstimulated (control) tissue. The samples are taken from slices that were stained with Iba1 (A), H&E (B), LFB (C), and NeuN (D), respectively. The scale bars represent 100 *μ*m. Note that, in these samples, the observed damage can only be attributed to the implantation, residence, or explantation of the array. (E)–(H) Samples near electrode tips that were subjected to 20 *μ*A stimulation. Duty cycles were all 1/3; stimulation durations were 1 s (E), 1 s (F), 5 s (G), and 1 s (H). The tissue samples are taken from slices that were stained with Iba1 (E), H&E (F), LFB (G), and NeuN (H). (I)–(L) Samples near electrode tips that were subjected to 100 *μ*A stimulation. Duty cycles were 1/1 (I) and 1/3 ((J)–(L)). All stimulation durations were 1 s. The tissue samples are taken from slices that were stained with Iba1 (I), H&E (J&K), and LFB (L). The scale bar represents 100 *μ*m.

- Unstimulated electrodes (figures 5(A)–(D)): we identified 39 samples near unstimulated electrode tips across all six arrays: seven in slices stained with Iba1, nine with NeuN, 14 with H&E, and nine with LFB/CV. While no significant change (other than the presence of the electrode track) was typically seen, gliosis and/or astrocytic scarring was observed around the shaft or tip of a few electrodes. No reduction of the density of NeuN was observed near the tip.
- 10 *μ*A, 1/1 duty cycle, 5 s: four samples were identified in one array: two stained with Iba1, one with NeuN, and one with H&E. Iba1 antibody stains did not exhibit an increase near electrode tips. The H&E stain showed minimal non-specific damage to the tissue near an electrode tip. The NeuN-stained tissue did not show a qualitative reduction in neural density.
- 20 *μ*A, 1/1 duty cycle, 1 s (figures 5(E)–(H)): the one tissue sample that corresponded to this condition was stained with Iba1 antibody and exhibited no qualitative increase in antibody density.
- 20 *μ*A, 1/1 duty cycle, 5 s: seven samples were identified across two arrays: five in slices stained with the Iba1 antibody, one with NeuN, and one with H&E. An increase in Iba1 antibodies was only observed in one of the five sections. There was no qualitative reduction in the density of NeuN and the H&E stain showed no abnormalities.
- 20 *μ*A, 1/3 duty cycle, 1 s (figures 5(E)–(F), (H)): 15 samples were identified in one array: five stained with H&E, six with Iba1 antibody, and four with NeuN. The H&E stains show tissue adhesion to the electrodes, similar to other stimulation paradigms. Tissue in the Iba1 antibody-stained sections appeared healthy and NeuN stains did not indicate any qualitative reduction in the density of NeuN.
- 20 *μ*A, 1/3 duty cycle, 5 s (figure 5(G)): 15 cortical samples were identified within three arrays: five stained with H&E, six with LFB/CV, four with NeuN. There were no samples with Iba1 antibody treatment. Several sections with anatomical stains (H&E and LFB/CV) showed a small increase in gliosis and astrocytic scarring in the neuroparenchyma along the perimeter of electrode tracks. However, there was no increase in inflammation, and NeuN stains did not indicate a reduction in the number of NeuN around the electrode tips.
- 100 *μ*A, 1/1 duty cycle, 1 s (figure 5(I)): four samples were identified from one array, on slices stained with the Iba1 antibody. No significant increase in the presence of Iba1 antibodies was observed near any of the four electrode tips. However, there was significant tissue damage, likely due to the removal of the array because similar damage was observed on control electrodes.
- 100 *μ*A, 1/3 duty cycle, 1 s (figures 5(J)–(L)): four samples were identified in one array: 2 in slices stained with H&E, and two with LFB/CV. The H&E stains exhibited slight damage to tissue along the perimeter of the electrode tracks, whereas the LFB/CV stains did not exhibit such damage (figures 5(J)–(L)).

### Scoring of tissue damage

**Figure 6.**
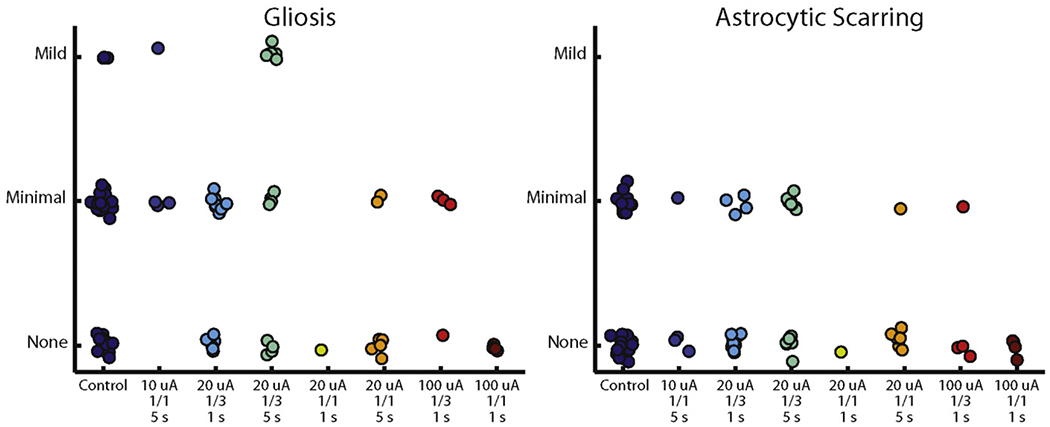
Distribution of scores for each sample. The only group whose score distribution was significantly different from the control condition was the 20 *μ*A, 1/3 duty cycle, 5 s condition (*p* = 0.03, Fisher–Freeman–Halton exact test).

The damage of the tissue around each electrode tip was also scored by the histopathologist (figure 6 and table 1). Statistical analysis revealed that only one regime—20 *μ*A, 1/3 duty cycle, 5 s—yielded a significantly different gliosis score compared to the control group (Fisher–Freeman–Halton exact test, *p* = 0.03). No groups showed a difference in astrocytic scarring or pigmentation. Even the strongest stimulation regimes (100 *μ*A) did not exhibit significant changes relative to control electrodes, and no relationship was observed between stimulation regime and score. This semi-quantitative analysis supports the claim that chronic ICMS does not produce any detectable tissue damage. It should be noted, however, that few samples were available for the most intense stimulation regime—namely 100 *μ*A—so results from this condition should be interpreted with caution.

### Quantitative comparison of neuronal density

**Figure 7.**
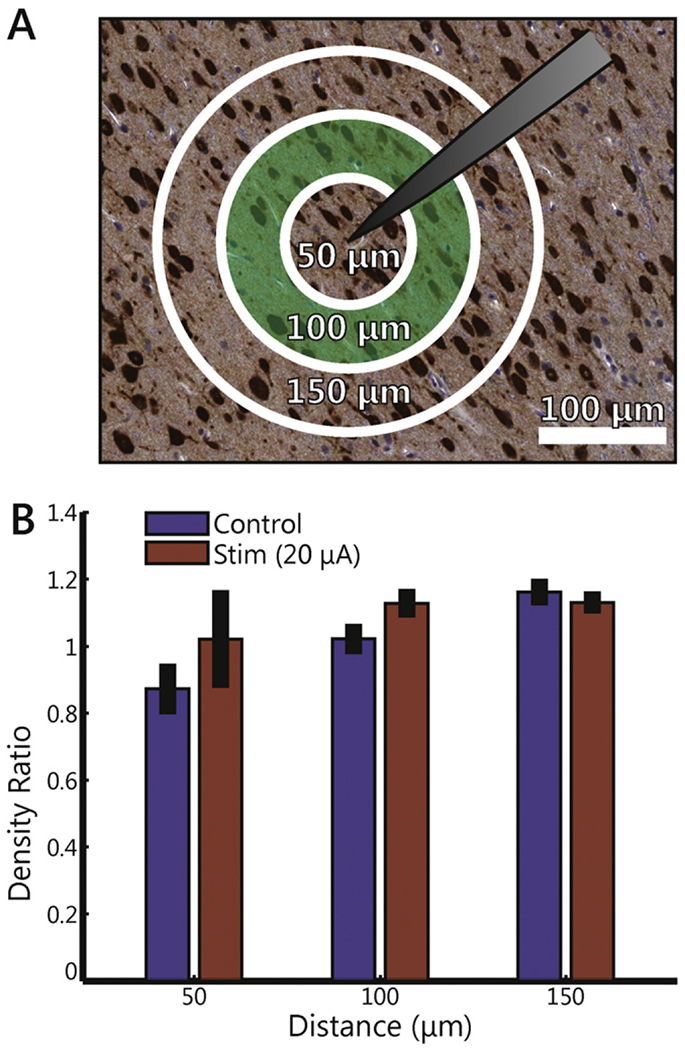
Quantitative analysis of neuronal density. (A) Example of tissue showing the concentric annuli of increasing radii. Sample shown was stimulated at 20 *μ*A. (B) Average normalized neuronal density for control tissue and tissue stimulated at 20 *μ*A within distances of 50, 100, and 150 *μ*m of the electrode tip. Error bars indicate standard error of the mean. Although there is a general (non-significant) trend of increased density as distance increases, there are no differences between control and stimulated tissue.

To validate the qualitative assessments of neuronal density provided by the histopathologist described above, we performed a quantitative analysis of density on the 20 *μ*A samples, comparing these to control samples. We did not observe significant differences in neuronal density between the stimulated and unstimulated tissue (figure 7(B)) (ANOVA: *F*(1, 48) = 0.3, *p* = 0.59). Further, there was no difference in density across tissues that were sampled at different distances (from 50 to 150 *μ*m) from the electrode tip (*F*(2, 48) = 0.7, *p* = 0.52), nor was there an interaction between stimulation condition and distance (*F*(2, 48) = 0.16, *p* = 0.85). While this analysis was performed with a limited number of samples (a total of 18), results support the conclusion of the qualitative histopathological analysis that neuronal density was not affected by stimulation.

### Effects on behavior

The daily assessment of behavior revealed no effect of stimulation on the NHPs’ ability to retrieve any of the treats presented, regardless of the size of the treat. Additionally, there were no noticeable abnormalities in the strategies used, preshaping, time to contact, or the accuracy and precision of retrieval for the duration of the implants. Ability to grasp or manipulate any of the presented treats was unimpaired, and the use of the hand contralateral to the stimulation appeared to be completely normal in these daily tests as well as in their behavior in the cage. In summary, neither the implantation process, the residence of the arrays, nor chronic ICMS produced detectable deficits in the animals’ ability to perform fine motor behaviors.

## Discussion

### Effect of array implantation and residence

Stimulation would be expected to result in ‘dose dependent’ effects on tissue: higher stimulation amplitudes and durations would cause greater neuronal necrosis, gliosis, atrophy, etc. In contrast, morphological changes that are apparent uniformly across the array, or that are limited to the superficial layers of cortex are unlikely to be caused by stimulation. Instead, these effects are likely to be caused by implantation of the array and its chronic residence in cortex.

According to the histopathologist, insertion and removal of the array likely caused most if not all of the tissue damage through shearing of tissue adhered to the electrodes and substrate. Tissue damage was observed both immediately deep to the array and along the shank of the electrodes and is consistent with the damage caused by arrays without any stimulation [35, 36, 38]. That the damage in control samples was comparable to that observed in stimulated samples further supports the conclusion that the observed damage is primarily attributable to the implantation, residence, and explantation of the electrode arrays.

Histologic changes were limited to cortex underneath the implanted arrays, and included cortical atrophy, gliosis, macrophage infiltration with pigmentation, insertion tracks from the arrays, and neuronal necrosis. All of the observed changes are consistent with limited damage caused by the initial implantation of the array, and minimal to mild ongoing degeneration resulting from the chronic residence of the array. The changes at depths near electrode tips were minimal, and there was no evidence of ongoing neuronal degeneration at these depths. Rather, changes occurred either throughout all layers, or were focused around superficial cortex, at or near the interface between the array and the cortical surface.

### Effects of stimulation parameters

#### Charge amplitude.

Stimulation amplitude, within the range delivered in this study, was not predictive of tissue health. The tissue surrounding the electrode tips that were subjected to the highest current amplitude (100 *μ*A) did not exhibit more damage than tissue surrounding the tips of electrodes that had not been stimulated or that had been subjected to lower currents. Unfortunately, the sample size of tissue samples near stimulated electrodes was small, and did not include every stain for every stimulation condition. The 100 *μ* A conditions in particular were underrepresented, especially compared to the 20 *μ* A conditions. Even so, in only one of the four 20 *μ* A stimulation condition (1/3 duty cycle, 5 s duration) did several samples exhibit noticeable changes compared to controls (along a single measure, namely gliosis, figure 6). Astrocytic scarring for the 20 *μ*A samples was equivalent to that of control samples, as was neuronal density. If charge amplitude (or charge density) were a determining factor in tissue health, other stimulation regimes with 20 *μ*A amplitude would have exhibited signs of tissue damage (not to mention the more intense stimulation regimes).

#### Duty cycle.

There was no evidence that varying duty cycle between stimulation regimes had any effect on cortical tissue. Indeed, at 20 *μ*A and 5 s duration, samples from the 1/3 duty cycle tended to exhibit greater tissue damage than samples from its 1 /1 counterpart, a paradoxical effect that is likely due to the small sample size of the 1/1 duty cycle condition (7 sections, compared to 15 sections for the 1/3 duty cycle).

#### Interval duration.

Stimulation duration may have had a small effect on tissue health. The largest observed difference was between the 20 *μ*A, 1/3 duty cycle, 1 s condition compared to the corresponding 5 s condition. The tissue that received the longer duration stimulation showed a significant increase in gliosis and astrocytic scarring. On the other hand, there was no difference in the NeuN densities between the two conditions, suggesting that the populations of neurons near these electrode tips were still healthy.

Overall, the histological analysis revealed that tissue surrounding stimulated and non-stimulated tissue exhibit similar attributes, suggesting that functionally relevant stimulation regimes do not cause a reduction in the density of healthy neurons. The only stimulation regime that yielded detectably greater gliosis and astrocytic scarring than unstimulated electrodes was the 20 *μ*A, 1/3 duty cycle, 5 s duration condition. However, gliosis and astrocytic scarring are expected biological responses to the presence of the electrodes in the neural tissue and vary widely even given the same electrode geometry [34, 35, 38] so the observed effect may have been an artifact of a small sample size. With more samples (15) for the two 20 *μ*A, 1/1 conditions, the small differences in tissue attributes between these two regimes may be evidence that stimulation duration may have a small effect on tissue damage. Overall, however, while individual conditions often had small sample sizes, we identified 40 sections that were near stimulated electrode tips, and another 39 that contained the tips of control electrodes. Over this large sample, the effects of ICMS were largely absent.

### Comparison with other studies

Previous studies have reported significant changes in neural tissue morphology and associated neural recording capacity due to chronic electrode array implantation and residence. A well-organized sheath of fibroblast-generated collagen has been shown to form around chronically implanted electrodes [36], which is thought to contribute to an increase in electrode impedance [36, 39] as observed changes in impedance tend to be larger to the extent that this sheath is present [39]. Increased impedance and tissue reactions also follow a similar time course: impedance ramps up for approximately one week then exhibits a slow decline [39] while the electrode-tissue interface stabilizes during the first two weeks [40]. The evolution of the impedance for the control electrodes of the present study has recently been documented to follow the typical time course [41].

In acute ICMS studies, microstimulation at amplitudes ranging from 25 to 140 *μ*A was found to induce no tissue damage or gliosis [42]. In a study on chronic ICMS, McCreery [30] stimulated electrodes for 7 h at 50 Hz (at 800 and 1600 *μ*C cm^−2^ or 52 and 104 nC ph^−1^ or 130–260 *μ*A) for 10 days and found that no neuronal damage was induced, but immune responses were triggered. However, it is important to note that the stimulation parameters used in this previous study (high amplitude, low frequency) were very different from those used in the present study. In another study, fibrous tissue was found to form around UEA electrodes after being stimulated for up to 3 months at 250 Hz and 100 *μ*A [27]. Using a quantitative approach to histology, McCreery and colleagues observed a loss of neurons within 60–150 *μ*m of electrode tips after stimulating at 50 Hz, 8 h a day for 30 days, with the extent of neuronal loss depending on the stimulation parameters [21]. Continuous stimulation at 10 *μ*A did not cause any discernible damage, as there was no difference in neural density around electrodes stimulated at that amplitude compared to unstimulated electrodes. Stimulation at 20 *μ*A, with a 1/1 duty cycle and 1-s pulse trains, induced a reduction in neuronal density that was greater within 60 *μ*m of stimulated electrode tips. When stimulation was continuous rather than intermittent, the radius of damaged tissue increased to 150 *μ*m. At an equivalent amplitude, we did not observe such significant neuronal damage, suggesting that continuous stimulation contributed to the damage in the previous study. However, we too found that longer pulse trains tended to produce more gliosis than shorter ones (among the 20 *μ*A stimulation regimes, at least).

Finally, that our pulses were anodal phase-leading rather than the standard cathodal phase-leading might account for some of the differences between our results and those of previous studies. Indeed, stimulation with cathodal phase-leading is more effective at eliciting percepts than is stimulation with reversed polarity [43–45]. One possibility is that the efficiency of cathodal phase-leading stimulation is accompanied by an increased tendency to damage the neuronal tissue.

### Consequences of chronic ICMS on electrode performance

Despite the changes to the neural substrate caused by implantation, recordings have been successfully obtained with UEAs even after months of ICMS, indicating the presence of functional neurons in close proximity to electrode tips. Rousche and Normann [27] found that multiple stimulation sessions did not significantly alter the signal to noise ratio or the number of recordable units. Parker and colleagues found that stimulated and non-stimulated arrays exhibited comparable declines in the number of action potentials recorded [17]. In fact, under certain circumstances and for short periods of time, stimulation may reverse the impedance drops due to tissue encapsulation [46] thereby actually improving single-unit recordings [47].

Further, stimulation seems to remain effective despite any changes to neural tissue and reductions in the quality of neural recordings that may occur. First, deep brain stimulation has been found to be effective for years [48], although the surface area of these electrodes is many times larger than the microelectrodes investigated here. Second, Parker and colleagues were able to evoke EMG responses after months of ICMS [17]. Third, studies have shown that ICMS applied to primary sensory areas can evoke percepts over months or years [18, 27, 49–51].

In summary, studies of chronic ICMS have reported three main findings: both implanted and stimulated tissue react to the electrodes in a similar way; the quality of single-unit recordings usually decreases over time; despite damage to the neural tissue, the ability to stimulate is maintained over long periods with, in some cases but not others, small but progressive decreases in sensitivity to ICMS.

### Consequences of chronic ICMS on fine motor control

Critically, any damage that may have resulted from the implantation of and stimulation through the electrodes did not result in behavioral deficits in our subjects. Previous studies with primates have explored the consequences of chemical inactivation of S1 [52] and of surgically induced microlesions in S1 [32] on performance in prehension tasks. Both studies reported profound sensory and motor impairments, as evidenced by discoordination of finger movements, misapplied forces, and the use of alternate movement strategies. These studies demonstrate that fine motor tasks are sensitive to small lesions in S1. While it is possible that the behavioral assays used in this study were insufficiently sensitive to detect deficits in fine motor control, at the very least they show that no coarse motor impairment is caused by chronic ICMS. That the NHPs in this study were completely unimpaired in this fine motor task supports the conclusion that chronic ICMS produces only minimal damage in S1 if any.

## Conclusions

Our results suggest that chronic ICMS causes minimal tissue damage over a wide range of stimulation conditions and does not result in any detectable deficits in fine sensorimotor behavior. The stimulation parameters used in the present study have been shown to elicit meaningful percepts [6, 7]. Thus, our findings suggest that ICMS from chronically implanted microelectrode arrays may be a viable approach to convey sensory feedback in upper limb neuroprostheses.

## Figures and Tables

**Table 1.** Number of tissue samples near identified electrode tips that received each score given by the histopathologist, who had been blinded to the stimulation condition. The only group whose score distribution was significantly different from the control condition was the 20 *μ*A, 1/3 duty cycle, 5 s condition (*p* = 0.03, Fisher–Freeman–Halton exact test).

	Amp. (*μ*A)		10	20	20	20	20	100	100
	Duty cycle	Control	1/1	1/3	1/3	1/1	1/1	1/3	1/1
	Duration (s)		5	1	5	1	5	1	1

	No damage	25	3	11	6	1	6	3	4
Scarring	Minimal	11	1	4	8	0	1	1	0
	Mild	0	0	0	0	0	0	0	0

	No damage	15	0	6	4	1	5	1	4
Gliosis	Minimal	18	3	9	4	0	2	3	0
	Mild	3	1	0	6	0	0	0	0
